# Molecular-Biology-Driven Treatment for Metastatic Colorectal Cancer

**DOI:** 10.3390/cancers12051214

**Published:** 2020-05-13

**Authors:** Eleonora Lai, Nicole Liscia, Clelia Donisi, Stefano Mariani, Simona Tolu, Andrea Pretta, Mara Persano, Giovanna Pinna, Francesca Balconi, Annagrazia Pireddu, Valentino Impera, Marco Dubois, Marco Migliari, Dario Spanu, Giorgio Saba, Silvia Camera, Francesca Musio, Pina Ziranu, Marco Puzzoni, Laura Demurtas, Valeria Pusceddu, Manuela Dettori, Elena Massa, Francesco Atzori, Mariele Dessì, Giorgio Astara, Clelia Madeddu, Mario Scartozzi

**Affiliations:** 1 Medical Oncology Unit, University Hospital and University of Cagliari, 09042 Cagliari, Italy; ele.lai87@gmail.com (E.L.); nikina310788@gmail.com (N.L.); cleliadonisi@gmail.com (C.D.); mariani.step@gmail.com (S.M.); simo.tolu@tiscali.it (S.T.); an.pretta@gmail.com (A.P.); marapersano@alice.it (M.P.); giovannapinna91@gmail.com (G.P.); frabalconi@gmail.com (F.B.); pireddu.annagrazia@tiscali.it (A.P.); vola_90@live.it (V.I.); marco.dubois92@gmail.com (M.D.); m.migliari92@gmail.com (M.M.); dario.spanu@gmail.com (D.S.); sabagiorgio@live.it (G.S.); silvia.camera@hotmail.it (S.C.); francesca.musio@gmail.com (F.M.); pziranu@libero.it (P.Z.); marcopuzzoni@gmail.com (M.P.); lau.demi81@gmail.com (L.D.); valeria.pusce@gmail.com (V.P.); dinetto13112012@gmail.com (E.M.); francescoatzori74@yahoo.it (F.A.); marieledessi@tiscali.it (M.D.); giorgioastara@virgilio.it (G.A.); clelia_md@yahoo.it (C.M.); 2 Medical Oncology Unit, Sapienza University of Rome, 00161 Rome, Italy; 3 Medical Oncology Unit, Azienda Ospedaliera Brotzu, Ospedale Businco, 09134 Cagliari, Italy

**Keywords:** metastatic colorectal cancer, molecular biomarkers, therapeutic implications, tailored treatment

## Abstract

Background: Metastatic CRC (mCRC) is a molecular heterogeneous disease. The aim of this review is to give an overview of molecular-driven treatment of mCRC patients. Methods: A review of clinical trials, retrospective studies and case reports was performed regarding molecular biomarkers with therapeutic implications. Results: *RAS* wild-type status was confirmed as being crucial for anti-epidermal growth factor receptor (EGFR) monoclonal antibodies and for rechallenge strategy. Antiangiogenic therapies improve survival in first- and second-line settings, irrespective of *RAS* status, while tyrosine kinase inhibitors (TKIs) remain promising in refractory mCRC. Promising results emerged from anti-HER2 drugs trials in HER2-positive mCRC. Target inhibitors were successful for *BRAF^V600E^* mutant mCRC patients, while immunotherapy was successful for microsatellite instability-high/defective mismatch repair (MSI-H/dMMR) or DNA polymerase epsilon catalytic subunit (*POLE-1*) mutant patients. Data are still lacking on *NTRK, RET, MGMT*, and TGF-β, which require further research. Conclusion: Several molecular biomarkers have been identified for the tailored treatment of mCRC patients and multiple efforts are currently ongoing to increase the therapeutic options. In the era of precision medicine, molecular-biology-driven treatment is the key to impro patient selection and patient outcomes. Further research and large phase III trials are required to ameliorate the therapeutic management of these patients.

## 1. Introduction

Colorectal cancer (CRC) is the second most common tumor worldwide and the second leading cause of cancer death [1]. Approximately 20% of CRC patients present metastases at the time of diagnosis, and an additional 20% of them develop metastases during follow-up and need systemic therapy. Over the last years, robust research demonstrated that metastatic CRC (mCRC) is not one single entity, but rather a complex disease characterized by significant molecular heterogeneity [2]. In the era of precision medicine, it is crucial to identify predictive biomarkers to tailor treatment and to identify the patients who are more likely to respond to specific therapeutic agents. Several molecular biomarkers and related pathways have been investigated as potential targets, and consequently a wide variety of drugs have been developed, including monoclonal antibodies (mAbs), small molecule tyrosine kinase inhibitors (TKIs), and immune checkpoint inhibitors (ICI) [3,4]. 

The aim of this review is to give an overview of molecular-driven treatment of mCRC, focusing on already known molecular biomarkers and their impact on treatment decision-making, as well as new promising biomarkers with potential therapeutic implications.

## 2. Molecular Targets and Clinical Trials

**Figure 1:**
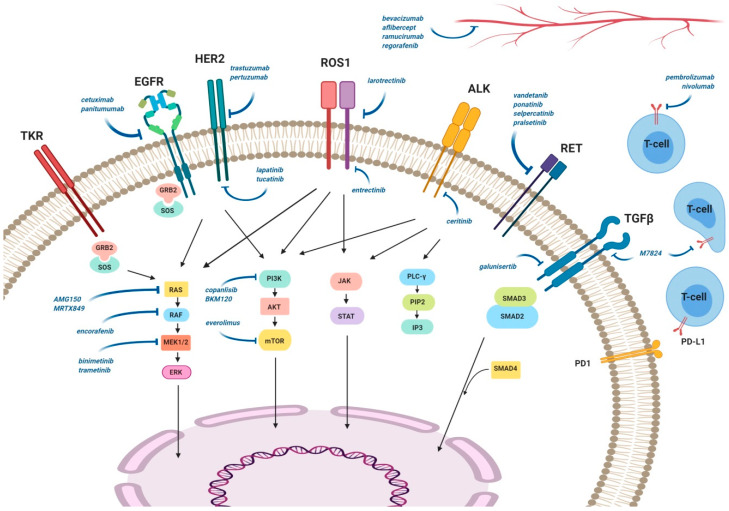
Potential targets and drugs for molecular-biology-driven treatment for metastatic colorectal cancer.

In the era of precision oncology, targeted therapy allows tailored treatment of mCRC patients and improvement of their recruitment in clinical trials, with selection being made according to specific molecular tumor characteristics (Figure 1). 

### 2.1. Epidermal Growth Factor (EGF)/EGF Receptor (EGFR) Pathway

The EGF/EGFR pathway plays a key role in tumor progression through regulation of proliferation, survival, angiogenesis, invasion, and immune evasion. EGFR is a glycoprotein that belongs to the erythroblastosis oncogene B (ErbB) family, composed of four related receptors of tyrosine kinase (RTKs): EGFR1 (also called EGFR), ErbB2 (HER2/neu), ErbB3 (HER3), and ErbB4 (HER4) [5]. In the absence of its specific ligands, EGFR remains in a state of inhibition [6]. When a ligand binds to the extracellular domain of EGFR, it induces homo- or heterodimerization with the other RTKs of the family, trigging ATP-dependent phosphorylation of the intracellular tyrosine kinase domain. This activates signal transduction to the cytoplasm and then to the nucleus, through the mitogen-activated protein kinase (MAPK) rat sarcoma (RAS)/rapidly accelerated fibrosarcoma proto-oncogene serine/threonine protein kinase (RAF)/mitogen-activated protein kinase (MEK)/extracellular signal-regulated kinase (ERK) pathway, which ultimately promotes tumor growth and progression [7]. Signal transduction through EGFR also activates the phosphatidylinositol-3-kinase (PI3K)/protein kinase B (AKT)/mammalian target of rapamicine (mTOR) signalling cascade, which is critical to cell survival, motility, and invasion, thus further promoting cancer survival and progression [8,9,10]. *EGFR* gene upregulation occurs in 30–70% of CRC [11], and its overexpression has been associated with metastatic risk [12]. Many studies have assessed the efficacy of anti-EGFR mAbs cetuximab and panitumumab as first-line treatments in *RAS* wild type (wt) mCRC and confirmed the mutational status of *RAS* as an independent predictive factor.

The ”Panitumumab Randomized trial In combination with chemotherapy for Metastatic colorectal cancer to determine Efficacy” PRIME study and the “Cetuximab Combined with Irinotecan in First-Line Therapy for Metastatic Colorectal Cancer” CRYSTAL study were the phase III trials, which demonstrated the efficacy of the combination of anti-EGFR plus chemotherapy versus chemotherapy alone. More specifically, the PRIME trial showed the superiority in progression-free survival (PFS) of oxaliplatin, 5-fluorouracil (5-FU) leucovorin, (FOLFOX) plus panitumumab versus FOLFOX (10 months vs. 8.6 months); overall survival (OS) was 23.9 months for the FOLFOX–panitumumab arm vs. 19.7 months for the FOLFOX arm [13,14]. In the CRYSTAL trial, irinotecan, 5-FU, leucovorin, (FOLFIRI)–cetuximab reduced the risk of progression compared to FOLFIRI alone [15]. 

Some trials compared the association of chemotherapy with an anti-EGFR vs. the association of chemotherapy with the anti-vascular endothelial growth factor (VEGF) mAb bevacizumab as first-line treatments for mCRC; however, the studies were negative for their primary endpoints. The FIRE-3 was a randomized phase III trial that compared FOLFIRI plus cetuximab with FOLFIRI plus bevacizumab in Kirsten RAS oncogene homolog (*KRAS*) wt mCRC. In the final *RAS* wt population, median OS (mOS) with FOLFIRI–cetuximab was 33.1 months (95% confidence interval (CI):24.5–39.4) compared to 25 months (23.0–28.1) with FOLFIRI–bevacizumab (hazard ratio (HR): 0.70, 0.54–0.90; *p* = 0.0059), whereas objective response (OR) and PFS results were comparable [16]. In the phase II PEAK study, patients were randomized to receive either modified FOLFOX6 (mFOLFOX6)–panitumumab or mFOLFOX6–bevacizumab. The final analysis in *RAS/* v-raf murine sarcoma viral oncogene homolog B1 *(BRAF)* wt mCRC patients showed a median PFS (mPFS) of 13.1 months in the mFOLFOX6–panitumumab arm vs. 10.1 months in mFOLFOX6–bevacizumab arm; mOS was 41.3 vs. 28.9 months, respectively [17,18]. The Cancer and Leukemia Group B (CALGB 80405 trial investigated FOLFIRI or mFOLFOX6 (investigator’s choice) in combination with either cetuximab or bevacizumab in *KRAS* wt (codons 12 and 13) mCRC patients and showed no differences in OS (primary endpoint) between the treatment groups [19]. Recently, primary tumor sidedness emerged as a predictive factor for response to anti-EGFR treatment; in particular, left-sided tumors would benefit from anti-EGFR mAbs, whereas right-sided tumors are considered anti-EGFR-resistant [20]. Tumor sidedness might actually be a surrogate of biological or molecular aspects; however, further research is needed to prospectively validate its role, and each clinical case requires medical discussion.

In order to improve the efficacy of these agents, several strategies are under investigation, including the combination of anti-EGFR with triplet chemotherapy [21] or with new agents, and more recently anti-EGFR rechallenge (Table 1). The rationale of rechallenge is based on the possible clonal selection under the pressure of anti-EGFR or anti-VEGF treatment, and requires re-evaluation of *RAS/BRAF* mutational status in circulating tumor DNA (ctDNA) by liquid biopsy in mCRC patients with acquired resistance to prior chemotherapy plus anti-EGFR. The activity of retreatment with a cetuximab-based therapy was investigated with encouraging results by Santini et al. (overall response rate (ORR) = 53.8%; mPFS 6.6 months) [22], while a retrospective analysis of patients treated in PRIME and PEAK trials who were rechallenged with an anti-EGFR mAb showed a mOS of 14.2 months [23]. In the “Cetuximab Rechallenge in Irinotecan-pretreated Mcrc, *KRAS*, *NRAS* and *BRAF* wild type treated in 1st line with anti-EGFR Therapy” CRICKET study, cetuximab plus irinotecan were administered to 28 *RAS/BRAF* wt mCRC patients who had become resistant to these drugs in a first-line setting. The authors reported six partial responses (PR, 4 confirmed) and 9 stable disease (SD) responses (response rate (RR) 21%; 95% CI: 10–40%; disease control rate (DCR) 54%; 95% CI: 36–70%). *RAS* mutations were found in ctDNA collected at rechallenge baseline in 12/25 evaluable patients; no *RAS* mutations were detected in the case of PR. Additionally, mCRC patients who were *RAS* wt at ctDNA evaluation had significantly longer PFS than those with *RAS* mutation in ctDNA (mPFS 4.0 vs. 1.9 months; HR, 0.44; 95% CI, 0.18–0.98; *p* = 0.03) [24]. 

The “Rechallenge With Panitumumab Driven by RAS Dynamic of Resistance” CHRONOS study is ongoing (NCT03227926); it is a liquid-biopsy-driven trial to assess the efficacy of rechallenge with panitumumab in mCRC patients with ctDNA-proven *RAS*-mediated acquired resistance [25]. 

The rechallenge strategy appears to be very promising, as the secondary resistance to anti-EGFR mAbs represents a challenging issue in mCRC treatment and has not been fully understood yet. More recently, growing evidence has linked this phenomenon to the dynamic nature of tumor biology and to the CRC genetic heterogeneity. Indeed, *RAS* status is not fixed and it is dynamic over time. *KRAS* mutation occurrence is considered as a mediator of acquired resistance to EGFR blockade, while circulating cell-free DNA analysis is a useful tool used to detect this molecular alteration and monitor its status by repeating liquid biopsies over time [26]. 

Human epidermal growth factor receptor 2 (HER2) is a transmembrane RTK of the EGFR family. *HER2* gene amplification or somatic mutation is found in 7% of CRC patients and *HER2* positivity is more frequent in patients with *KRAS*, neuroblastoma *RAS* viral oncogene homolog (*NRAS*), or *BRAF* wt mCRC. *HER2* alterations were associated with anti-EGFR mAbs resistance in retrospective analyses in second- and third-line treatments [27,28,29]. In “HER2 Amplification for Colo-rectaL cancer Enhanced Stratification” HERACLES A (trastuzumab plus lapatinib), HERACLES B (pertuzumab plus trastuzumab-emtansine), Mountaineer (trastuzumab plus tucatinib), and “Multicenter Phase II study to evaluate efficacy and safety of combination therapy with trastuzumab and pertuzumab in patients with HER2-positive metastatic colorectal cancer” TRIUMPH (trastuzumab and pertuzumab) phase II trials, the HER2 blockade was effective and had a manageable safety profile in *HER2-*positive (*HER2+*) pretreated mCRC patients (Table 2) [30,31,32,33]. In the TRIUMPH study, clonal ctDNA mutations in *KRAS*, *BRAF*, phosphatidylinositol-4,5-bisphosphate 3-kinase catalytic subunit alpha (*PIK3CA*), or *ERBB2* (observed only in case of PD) appeared as possible predictive factors for primary resistance to anti-HER2 treatment [33]. Further investigation is required to assess optimal HER2 targeting in this setting.

### 2.2. VEGF–VEGF Receptor (VEGFR) Pathway

Angiogenesis is a complex mechanism which leads to the formation of new blood vessels from an existing vasculature. This process is strictly regulated by a balanced equilibrium between pro and antiangiogenic factors as well as multiple signalling pathways. Several actors are involved, the most important being the glycoproteins VEGF-A, VEGF-B, VEGF-C, VEGF-D, and placental growth factor (PIGF). During tumor growth, there is a balance disruption between inducer and inhibitor factors towards a proangiogenic signal to increase nutrient supply to the tumor. For these reasons, angiogenesis is considered to be one of the cancer hallmarks and plays a key role in CRC growth and metastatic spread [34,35,36,37,38,39,40,41,42,43,44,45,46]. VEGF and its receptors are highly expressed in human mCRC tissues and in tumor-associated endothelial cells, respectively [41,42]; however, the relationship between VEGF production in tumor tissue and its circulating levels is unclear, as well as its relationship with CRC outcomes. Some studies indicate VEGF and VEGFR as prognostic factors, showing an association between their overexpression and poor disease-free survival (DFS), poor OS, and early relapse [42,43,44]. Nevertheless, other studies have shown a non-significant prognostic value of VEGF [45]. Angiogenesis represents one of the most important therapeutic targets for mCRC treatment, and various strategies, including antiangiogenic mAbs and TKIs, have been investigated [47].

#### 2.2.1. Anti-Angiogenic mAbs

##### Bevacizumab

Bevacizumab is a humanized mAb blocking all isoforms of VEGF-A; several phase III trials demonstrated improved survival in combination with chemotherapy compared to chemotherapy alone in first-line and second-line settings, both in bevacizumab naive and pretreated patients, irrespective of tumor sidedness and *RAS/BRAF* mutational status [48,49,50]. Bevacizumab efficacy and safety have been assessed in first-line settings, both in association with intensive combination treatment, as with FOLFOXIRI in “Triplet plus bevacizumab” TRIBE [51,52] and TRIBE-2 [53] trials, and in combination with monochemotherapy in patients that were not candidates for intensive chemotherapy, as with trifluridine/tipiracil in the “open-label, randomised, non-comparative phase 2 study evaluating S 95005 (TAS-102) plus bevacizumab and capecitabine plus bevacizumab in patients with previously untreated metastatic COlorectal cancer who are non-eligible for intensive therapy-1 study” TASCO trial [54] and with capecitabine in the ”AVastin in the Elderly with Xeloda” AVEX trial for elderly patients [55]. Based on TASCO-1 results, the phase III, open-label SOLSTICE study (An open-label, randomised, phase III Study cOmparing trifLuridine/tipiracil (S 95005) in combination with bevacizumab to capecitabine in combination with bevacizumab in firST-line treatment of patients with metastatIC colorectal cancer who are not candidatE for intensive therapy) was designed, with the aim of demonstrating the superiority in terms of the PFS of the experimental arm (trifluridine/tipiracil + bevacizumab vs. capecitabine + bevacizumab) in the same patient setting (NCT03869892) [56]. 

Moreover, in the “Phase II Randomized, Double-Blind, Placebo-Controlled Study of Capecitabine Bevacizumab Plus Atezolizumab Versus Capecitabine Bevacizumab Plus Placebo in Patients With Refractory Metastatic Colorectal Cancer” BACCI: trial, the association of bevacizumab and capecitabine with or without atezolizumab in refractory mCRC patients has been evaluated, showing for the first time a PFS improvement with co-targeting programmed cell death protein 1 (PD-1)/ programmed death-ligand 1 PD-L1 and VEGF axes in mCRC [57]. The “randomized phase II study of folfoxiri plus Bevacizumab plus atezolizumab versus folfoxiri plus Bevacizumab as first-line treatment of unresectable Metastatic colorectal cancer patients” AtezoTRIBE (NCT03721653) is currently evaluating the efficacy in terms of the PFS of atezolizumab in combination with FOLFOXIRI–bevacizumab vs. FOLFOXIRI–bevacizumab as a first-line treatment in mCRC patients (Table 3).

##### Aflibercept

Aflibercept is a novel recombinant human fusion protein that acts as a decoy receptor, precluding the interaction of VEGF-A, VEGF-B, and PlGF with their receptors [58]. The safety and efficacy of second-line aflibercept plus FOLFIRI in mCRC patients after failure of an oxaliplatin containing regimen was demonstrated in the international phase III, randomized, double-blind “VEGF Trap (aflibercept) with irinotecan in colorectal cancer after failure of oxaliplatin regimen” VELOUR study [59]. This association improved both OS and PFS compared to FOLFIRI plus placebo (OS 13.5 vs. 12.6 months; PFS 6.9 vs. 4.67 months). Treatment-related adverse events (TRAEs) included grade 3 and 4 VEGF-specific events (arterial thromboembolism 1.8%, venous thromboembolism 7.9%, hemorrhage 2.9%, hypertension 19%) and chemotherapy-related events, such as diarrhea (19%), thrombocytopenia (3.3%), and febrile neutropenia (5.7%). These results were also confirmed in a study carried out on Japanese patients (mOS: 15.59 months; mPFS: 5.42 months) [60]. The same association appeared as an interesting option beyond second-line treatment in a study by Auvrai et al., in particular in antiangiogenic-free interval patients [61]. In the prospective stratified, biologically enriched phase II “seconD-line folfiri/aflIbercept in proSpecTIvely stratified, anti-EGFR resistaNt, metastatic coloreCTal cancer patIents with RAS Validated wild typE status” DISTINCTIVE study, *RAS* wt mCRC patients progressing after first-line treatment with oxaliplatin, fluoropyrimidines, and anti-EGFR mAbs receive second-line FOLFIRI–aflibercept and are prospectively allocated to either of two groups according to circulating VEGFR-2 levels at baseline [62]. One of the aims of the study is to prospectively validate VEGFR-2 plasma levels as a predictive factor for the efficacy of aflibercept plus FOLFIRI in the study population [62].

##### Ramucirumab

Ramucirumab is the most recent humanized anti-VEGFR-2 mAb, which demonstrated promising antitumor activity and a favorable toxicity profile in mCRC during phase I and II trials in association with standard chemotherapy. The double-blind, randomized phase III “Ramucirumab versus placebo in combination with second-line FOLFIRI in patients with metastatic colorectal carcinoma that progressed during or after first-line therapy with bevacizumab, oxaliplatin, and a fluoropyrimidine- RAISE trial” [63] compared the association of FOLFIRI–ramucirumab vs. FOLFIRI plus placebo in mCRC patients previously treated with bevacizumab, oxaliplatin, and a fluoropyrimidine. The study met both its primary endpoint (mOS 13.3 vs. 11.7 months) and its secondary endpoint (PFS,5.7 vs. 4.5 months), while ORR was equivalent in the two groups. Most common TRAEs were hematological events (38% vs. 24% grade 3 neutropenia), diarrhea, and stomatitis. Grade ≥3 hypertension was seen in 11% in the ramucirumab group vs. 3% in the placebo group. The most frequent low-grade hemorrhagic event was epistaxis, while the majority of grade ≥3 were gastrointestinal events; incidence of venous thromboembolic events was similar in both groups. A subgroup analysis demonstrated that the efficacy and safety of the association was independent of *KRAS* mutation status, time to progression (TTP, <6 versus ≥6 months), and age (<65 vs. ≥65 years) [64]. The results of the study conducted by Suzuki et al. are in line with these data and provided further information on the better efficacy of ramucirumab in bevacizumab-naïve patients compared to bevacizumab-pretreated subjects (mPFS of 8 months in bevacizumab-naive group vs. 5 months in bevacizumab-pretreated group; RR 23% vs. 3%; DCR 85% vs. 69%) [65]. A recent trial demonstrated that low relative dose intensity did not compromise the efficacy of ramucirumab plus modified FOLFIRI in mCRC patients, an interesting finding considering that chemotherapy dose modification is often required [66]. 

#### 2.2.2. TKIs

##### Regorafenib

Regorafenib is an oral first-generation TKI that inhibits angiogenic RTKs, such as VEGFR-1, 2, and 3; tyrosine kinase with immunoglobulin-like and EGF-like domains (TIE)29; stromal RTKs as platelet-derived growth factor receptor (PDGFR), fibroblast growth factor receptor (FGFR), and oncogenic RTK: v-kit Hardy-Zuckerman 4 feline sarcoma viral oncogene homolog (KIT); rearranged during transfection (RET); v-raf-1 murine leukemia viral oncogene homolog 1 (RAF-1); and BRAF [67]. In the CORRECT (“patients with metastatic COloRectal cancer treated with REgorafenib or plaCebo after failure of standard Therapy”) and its post hoc subgroup analysis of Japanese vs. non-Japanese patients, CONCUR (“Asian Subjects With Metastatic Colorectal Cancer Treated With Regorafenib or Placebo After Failure of Standard Therapy”), and CONSIGN (“Regorafenib for Patients With Metastatic Colorectal Cancer Who Progressed After Standard Therapy”) phase III clinical trials, regorafenib improved PFS and OS in combination with best supportive care (BSC) compared with BSC alone in patients with mCRC progressing to standard treatment [68,69,70,71]. The most common grade ≥3 TRAEs were of hand–foot skin reaction (HFS), fatigue, diarrhea, hypertension, and rash or desquamation. 

More recent studies have evaluated the association of regorafenib with other drugs after failure of standard therapies. A phase I study showed that the combination of 160 mg regorafenib and a standard dose of cetuximab had promising activity and was well tolerated [72]. Another phase I trial identified the recommended dose of 120 mg regorafenib in association with nivolumab. Grade ≥3 regorafenib-related adverse events occurred in 40% of patients, with the most common grade ≥3 events being skin rash (12%), proteinuria (12%), HFS (10%), and liver dysfunction (6%). Survival results are still expected [73]. 

Various TKIs other than regorafenib are under investigation for pretreated mCRC patients in order to improve the outcome in this setting, with some showing efficacy and an acceptable safety profile (Table 4) [74,75,76,77,78,79,80].

### 2.3. MAPK–RAS/RAF/MEK/ERK Pathway 

MAPK signaling is characterized by a sequential activation of kinases, which finally leads to regulation of cell proliferation, differentiation, and death [81]. The MAPK pathway has been identified as one of the most strongly associated gene markers of CRC from a genome-wide association study conducted in Germany [82]. Somatic mutations in MAPK were shown to be correlated with poor survival after CRC diagnosis in a study by Barault et al. *KRAS* and *NRAS* activating missense mutations have been reported in 40% and 4% of CRC, respectively; up to 95% of mutations involve one of three major hotspots (residues G12, G13, and Q61) [83]. As for elderly patients, a different incidence of *KRAS* mutation has been reported according to the microsatellite status of the tumor; indeed, in this population, *KRAS* mutation seems to be more frequent in the case of microsatellite-stable MSS CRC, especially in males, whereas it seems to be lower in microsatellite-unstable tumors or with high mutational burden [84,85]. 

*RAS* has been long considered to be “undruggable”, but in 2017 Zeng et al. described potential strategies to target the mutated cysteine in *KRAS^G12C^* [86]. The first KRAS^G12C^ inhibitor in clinical development is AMG 510, a novel, first-in-class, small acrylamide-based molecule that specifically and irreversibly inhibits *KRAS^G12C^* by permanently locking it in an inactive guanosine diphosphate (GDP)-bound state through the binding to a pocket (P2) adjacent to the mutant cysteine of KRAS^G12C^. In preclinical studies, AMG 510 led to the regression of *KRAS^G12C^* tumors and to the development of a proinflammatory tumor microenvironment in immunocompetent mice, providing durable cures as a single agent and in improving the efficacy of other anticancer agents. Furthermore, in clinical trials, AMG 510 demonstrated antitumor activity in the first dosing cohorts [87]. A first-in-human, open-label, multicenter ongoing phase I study is evaluating the safety, tolerability, pharmacokinetics, and efficacy of AMG 510 in adult patients with locally-advanced or metastatic *KRAS^G12C^* mutant solid tumors; SD was achieved in 4/19 mCRC patients (NCT03600883) [88,89]. AMG 510 was well tolerated, with no dose limiting toxicities at studied doses; further data are expected. A phase I/II multiple expansion cohort trial is assessing MRTX849, another KRAS G12C inhibitor, in patients with advanced solid tumors with *KRAS^G12C^* mutation (NCT03785249). Confirmed PR has been reported in 1 mCRC patient [90]. 

The *BRAF* gene encodes a serine–threonine kinase, located immediately downstream of the *RAS* gene in the MAPK signalling, and it is mutated in about 5–10% of mCRC patients. The most frequent *BRAF* mutation is V600E (*BRAF^V600E^*), which is determined by single amino acid substitution at codon 600 of exon 15 in chromosome 7, with replacement of valine for glutamic acid. As a consequence, BRAF proteins acquire an active kinase conformation without dimerization and act as RAS-independent monomers, leading to the uncontrolled activation of the MAPK pathway. The mCRC patients with *BRAF^V600E^* mutation have a poor prognosis and a more aggressive disease [91]. Many studies are trying to develop new therapeutic strategies to increase survival in *BRAF* mutant mCRC patients. The “Binimetinib, Encorafenib, and Cetuximab Combined to Treat BRAF-Mutant Colorectal Cancer” BEACON CRC trial was a randomized, open-label, 3-arm phase III global study assessing triplet therapy with encorafenib (BRAF inhibitor), binimetinib (MEK inhibitor), and cetuximab or doublet with encorafenib plus cetuximab vs. irinotecan plus cetuximab or FOLFIRI plus cetuximab in *BRAF^V600E^* mutant mCRC patients progressing after one or two previous regimens. The mOS was 9.0 months in the triplet group and 5.4 months in the control group (HR=0.52; 95% CI: 0.39–0.70; *p* < 0.001). The confirmed RR was 26% (95% CI: 18–35) in the triplet group and 2% (95% CI: 0–7) in the control group (*p* < 0.001). The mOS in the doublet group was 8.4 months (HR vs. control, 0.60; 95% CI: 0.45–0.79; *p* < 0.001). The most common TRAEs in the triplet therapy were diarrhea, nausea, vomiting, and acneiform dermatitis. MEK inhibitor class-related TRAEs, including serous retinopathy and left ventricular dysfunction, occurred at previously described similar rates. Grade ≥3 TRAEs were observed in 58% of patients in the triplet group, in 50% in the doublet therapy group, and in 61% in the control group [92]. Patients treated in the experimental arms had a 44% reduction in the risk of quality of life (QOL) deterioration compared to control arm patients and no overall differences in QOL between triplet and doublet therapies were reported [93]. 

The combination of dabrafenib (BRAF inhibitor) and trametinib (selective MEK inhibitor) was evaluated in 43 patients with *BRAF* mutant mCRC. ORR was achieved in 12% of patients (including one CR), with a duration of response of over 36 months; SD was reported in 56% of patients. Reduced levels of phosphorylated ERK compared to baseline were shown in nine biopsies performed during treatment. Mutational analysis revealed that *PIK3CA* mutations did not preclude treatment response and neither *PTEN* loss nor MSI correlated with efficacy. The most frequent TRAEs were nausea, pyrexia, and fatigue [94]. An open-label phase I study was designed to investigate the safety, pharmacokinetics, pharmacodynamics, and clinical activity of trametinib and dabrafenib when administered in combination with panitumumab in patients with *BRAF^V600E^* mutant mCRC (NCT01750918). In the initial dose-escalation study, patients (*n* = 142) were enrolled to receive dabrafenib + panitumumab, dabrafenib + panitumumab + trametinib, and trametinib + panitumumab in order to identify the optimal dosing. Subsequently, expansion cohorts investigated the safety and clinical activity of each combination. Confirmed RR for dabrafenib + panitumumab, dabrafenib + panitumumab + trametinib, and trametinib + panitumumab were 10%, 21%, and 0%, respectively. Increased MAPK suppression was correlated with triplet efficacy and serial cell-free DNA analysis revealed *KRAS* and *NRAS* mutations at PD [95]. 

Several trials have been conducted and others are ongoing to investigate the role of target drugs in the MAPK pathway (Table 5). 

### 2.4. PI3K-AKT-mTOR Pathway

The PI3K/Akt/mTOR signaling axis plays a central role in regulating cellular metabolism, cytoskeletal reorganization, cell growth, and differentiation in response to different signals conveyed by RTKs and G-protein-coupled receptors [96,97,98]. Recent data revealed that the PI3K/AKT/mTOR cascade is implicated in CRC development and that its components are overexpressed in CRC [99]. *PI3K* mutations (especially on the PI3K catalytic subunit alpha *PIK3CA*) are reported in approximately 7–12% of CRC patients and may lead to upfront EGFR resistance [100,101,102,103,104,105]. In a meta-analysis of four trials, the rate of *PTEN* loss among 231 primary tumors was 38%, while the ORR was 6% vs. 32% with anti-EGFR mAbs in patients with and without *PTEN* loss, respectively [106]. Everolimus at 70 mg/week or 10 mg/day was evaluated in a phase II trial of mCRC patients previously treated with bevacizumab, fluoropyrimidine, oxaliplatin, and irinotecan-based regimens; it was well tolerated but did not show great efficacy [107].

A phase I trial investigated the feasible doses of weekly everolimus and irinotecan given with cetuximab for mCRC progression after 5-FU or capecitabine + oxaliplatin. However, the trial was terminated early because of clinical practice changes and emerging data on everolimus dosing [108]. An ongoing phase I trial is assessing the side effects and the best dose of the combination of cetuximab with everolimus in mCRC and head and neck cancer (NCT01637194). A phase Ib study investigated the association of panitumumab with BKM120, a PI3K inhibitor, in *KRAS* wt mCRC, but unfortunately there was little evidence of activity [109]. Finally, the association of the PI3K inhibitor copanlisib with nivolumab is still under evaluation (NCT03502733). 

### 2.5. Gene Fusions

#### 2.5.1. RET

RET is a transmembrane RTK for the glial-derived neurotrophic factor family [110]. As a consequence of *RET* gene fusions, chimeric RET proteins have a ligand-independent activation, leading to sustained activation of downstream survival and growth pathways, such as MAPK and PI3K/AKT/mTOR [111,112]. *RET* fusion occurs in 0.2% of solid tumors without concurrent driver mutations; the effect of *RET* activation is less clear in CRC, but several studies suggest that RET chimeric proteins might be associated with worse prognosis, poor treatment response, and reduced OS [113]. Due to the rarity of CRC, it is not easy to conduct clinical trials in this specific disease and data derive mainly from early trials or case reports. 

In patient-derived tumor cells (PDCs) with nuclear receptor coactivator 4 (NCOA4)-RET fusion obtained from the brain metastasis of a 63-year-old mCRC patient, vandetanib revealed a significant antitumor effect in terms of cell viability to CRC-PDCs [110]. Ponatinib, another TKI, was tested in *RET* fusion-positive CRC patient-derived xenograft (PDX) models compared to the standard of care agent 5-FU and demonstrated greater efficacy in the NCOA4-RET model (79% of tumor growth inhibition) and in the CCDC6-RET model (almost complete regression), identifying this drug as the most potent RET inhibitor tested [113]. Additionally, some trials are ongoing. The “Phase 1/2 Study of Oral LOXO-292 in Patients With Advanced Solid Tumors, Including RET Fusion-Positive Solid Tumors, Medullary Thyroid Cancer, and Other Tumors With RET Activation” LIBRETTO-001 trial is an open-label, first-in-human study that was designed to evaluate the safety, tolerability, pharmacokinetic, and preliminary antitumor activity of selpercatinib (LOXO-292), an oral RET kinase inhibitor, in advanced refractory solid tumor patients, including *RET* fusion-positive mCRC (NCT 03157128). The ARROW trial is a phase 1/2 study of the highly-selective RET inhibitor pralsetinib (BLU-667) in patients with thyroid cancer, non-small-cell lung cancer, and other advanced solid tumors comprised of mCRC (NCT03037385). 

#### 2.5.2. NTRK, Anaplastic Lymphoma Kinase (ALK), ROS Proto-Oncogene 1, Receptor Tyrosine Kinase (ROS1)

The tropomyosin receptor kinase (TRK) family activates several downstream signaling pathways, including MAPK, PI3K/AKT, or phospholipase C (PLC)-γ/protein kinase C (PKC), thus resulting in regulation of cellular proliferation or apoptosis [114]. *NTRK* gene fusion is uncommon in CRC (0.5–2.0%); some studies reported *TPM3-NTRK1* gene rearrangements [115,116], which result in constitutive dimerization of the chimeric protein that leads to ligand-independent activation of the TRKA kinase domain [117]. The presence of *TPM3-NTRK1* remains a rare event in CRC, but its occurrence is clinically significant, easily detectable by immunohistochemistry (IHC), and it might identify a subset of patients highly sensitive to TRK inhibitors.

Echinoderm microtubule-associated protein-like 4 (*EML4*)–*ALK* gene fusions were found in 2.4% of CRC specimens through exon array profiling [118]. A study reported the presence of rearrangement-positive cases for both *ALK* and *ROS1* in human CRC specimens and was the first to demonstrate a similarly low but detectable rate of ROS1 rearrangement in CRC [119]. Pietrantonio et al. found that *ALK, ROS1*, and *NTRK* fusions occurred more frequently in elderly patients with right-sided, node-spreading, *RAS* wt, MSI-high (MSI-H) mCRC, and were associated with shorter OS and poor prognosis [120]. 

Ceritinib, an ALK inhibitor, provided a significant clinical benefit in a refractory mCRC patient with a striatin (*STRN*)*–ALK* gene fusion, with a marked size decrease of skin metastasis and resolution of all contrast-enhancing tumors in a computed tomography scan [121]. Entrectinib is an orally pan-TRK, -ROS1, and -ALK inhibitor that is clinically active in patients with *NTRK-*rearranged tumors and has the ability to penetrate the blood–brain barrier [122]. Since the rarity of this fusion precludes clinical studies in a large series, specific data of entrectinib in mCRC derive mainly from case reports [123]. An integrated database included the pivotal data of three phase I or II clinical trials: “First-in-human, phase I study of entrectinib – an oral pan-trk, ROS1, and ALK inhibitor – in patients with advanced solid tumors with relevant molecular alterations”- ALKA-372-001, “Study of Oral RXDX-101 in Adult Patients With Locally Advanced or Metastatic Cancer Targeting NTRK1, NTRK2, NTRK3, ROS1, or ALK Molecular Alterations”- STARTRK-1, and “Basket Study of Entrectinib (RXDX-101) for the Treatment of Patients With Solid Tumors Harboring NTRK 1/2/3 (Trk A/B/C), ROS1, or ALK Gene Rearrangements (Fusions)”- STARTRK-2). These studies enrolled patients with locally advanced or metastatic *NTRK* fusion-positive solid tumors that were naïve to specific prior TKI to receive entrectinib. Results showed durable and clinically meaningful responses and a manageable safety profile [124]. Larotrectinib, a highly potent and selective ATP-competitive inhibitor of all three TRK kinases, demonstrated a centrally confirmed ORR of 75% in phase I and II clinical trials, with durable responses irrespective of age, histology, and specific fusion partner and without serious side effects in *NTRK* fusion-positive metastatic solid tumors [125]. These data suggest that the routinary test of *NTRK* fusions may increase the number of available therapeutic options.

### 2.6. MSI, POLE-1 Mutations, and Immunotherapy

#### 2.6.1. MSI

MSI are short repeated DNA sequences, which can become longer or shorter and make DNA unstable in the case of a defective mismatch repair system (dMMR). MSI is present in about 95% of Lynch syndrome and hereditary CRC cases, but can also be observed sporadically (15%) as a result of biallelic inactivation of mutL homolog 1 (MLH1) by the promoter hypermethylation [126]. MSI tumors have increased immunogenicity and high number of activated tumor infiltrating lymphocytes (TILs), primarily CD8+ cytotoxic T lymphocytes, CD4+ T helper 1 cells, and natural killer cells [127,128], which are considered to be predictive for response to anti-PD1/PD-L1 agents.

#### 2.6.2. DNA Polymerase Epsilon, Catalytic Subunit (POLE) 

The *POLE* gene, located in 12q24.33, encodes for the DNA polymerase epsilon catalytic subunit. *POLE* has an essential role in chromosomal DNA replication, in the synthesis of the main strand, and in recognition and removal of unpaired nucleotides. Several papers have identified alterations in the *POLE* catalytic subunit in about 3% of sporadic CRCs that were MSS but hypermutated. Stenzinger et al. detected somatic *POLE* exonuclease domain up to 12.3% of MSS sporadic CRC [129,130,131]. In fact, MSS tumors with *POLE* mutation can exhibit high loads of neoantigens and lymphocytes infiltrating both the tumor and its microenvironment. Gene expression profiling analysis documented the prevalence of *PD-L1* and *PD-1* gene expression levels in *POLE* hypermutant tumors compared with non-hypermutant tumors, suggesting that *POLE* hypermutant tumors may be good candidates for immunotherapy [132,133]. The analysis of *POLE* deficiency might then help in guiding personalized management of mCRC, in particular for tumors with proficient MMR (pMMR). 

#### 2.6.3. Immunotherapy

ICIs were explored in mCRC patients with MSI status and *POLE* mutations.

In the KEYNOTE-164 study, pembrolizumab was administered to 124 MSI-H/dMMR mCRC patients for up to 2 years until PD, unacceptable toxicity, or withdrawal. ORR was 33% (95% CI: 21–46) in cohort A (≥2 prior lines of standard therapy) and 33% (95% CI: 22–46) in cohort B (≥1 prior line of therapy); median duration of response was not reached in either cohort. The mPFS was 2.3 months (95% CI: 2.1–8.1) and 4.1 months (95% CI: 2.1–18.9). The mOS was 31.4 months (95% CI: 21.4-not reached) in cohort A and was not reached (95% CI: 19.2-not reached) in cohort B. Grade 3 and 4 TRAEs (pancreatitis, fatigue, increased alanine aminotransferase, and lipase) were reported in 16% of patients in cohort A and in 13% of patients in cohort B. Globally, has been pembrolizumab shown to be effective, with a manageable safety profile in this patient setting [134]. 

In the Check-Mate 142 trial, nivolumab provided durable responses and disease control, as well as long-term survival in pretreated patients with MSI-H/dMMR mCRC. Out of 74 patients enrolled, 54.1% had received ≥3 prior therapies. At a median follow-up of 12.0 months, 23 of 74 patients (31.1%) achieved investigator-assessed ORR; 68.9% (95% CI: 57.1–79.2) of patients had disease control for ≥12 weeks. Median duration of response was not reached; all responders were alive, and 8 (34.8%) had responses of ≥12 months. The most common TRAEs were fatigue (21.6%), diarrhea (20.3%), pruritus (13.5%), and rash (10.8%); grade ≥3 increased lipase (8.1%) and amylase (2.7%) levels were reported [135]. The updated results of the non-pretreated MSI-H/dMMR mCRC cohort were recently presented. MSI-H/dMMR mCRC patients received first-line nivolumab plus low-dose ipilimumab until PD or discontinuation. At a median follow-up of 13.8 months, for all 45 patients, ORR (primary endpoint) was 60% (95% CI: 44.3–74.3). Responses were consistent across subgroups, including age, Eastern Cooperative Oncology Group performance status, prior adjuvant or neoadjuvant therapy, and mutational status. Grade ≥3 TRAEs occurred in 16% of patients. Therefore, nivolumab plus low-dose ipilimumab demonstrated robust and durable clinical benefit and was well tolerated and might represent a new first-line treatment option for patients with MSI-H/dMMR mCRC [136,137]. 

Considering the rarity of the *POLE* mutation, almost all data derive from case reports. The first case in the literature described a clinical response to pembrolizumab in an 81-year-old man with treatment-refractory MSS mCRC with *POLE* mutation identified through genomic profiling with next-generation sequencing [138]. In the study conducted by Fargò et al., one *POLE* mutant mCRC patient received pembrolizumab and showed a marked and sustained response at 32 months of follow-up as assessed by positron emission tomography–computed tomography imaging, as well as decreased carcinoembryonic antigen levels [139]. Currently, various phase II studies with ICIs are ongoing and hopefully will provide new evidence for this mCRC subgroup (Table 6).

### 2.7. Transforming Growth Factor-β (TGF-β) Pathway

According to the CRC consensus molecular subtypes (CMS) classification, 23% of CRCs are classified as CMS4 (mesenchymal), which is characterized by prominent activation of TGF-β, stromal invasion, and angiogenesis [2,140]. Mothers against decapentaplegic homolog 4 (SMAD4) is the central mediator of TGF-β signaling; its mutations have been identified in CRC and it is associated mainly with colon cancer (rather than rectal cancer), female sex, and shorter OS than wt SMAD4 CRC patients (mOS 29 months vs. 56 months) [141]. The “LY3200882 and Capecitabine in Advanced Resistant TGF-beta Activated Colorectal Cancer - European Organisation for the Research and Treatment of Cancer 1615” EORTC1615 MoTriColor (Molecularly guided Trials with specific treatment strategies in patients with advanced newly molecular defined subtypes of Colorectal cancer) trial is a phase I/II study evaluating galunisertib (LY2157299), a small oral antagonist of the TGF-β receptor type 1 (TGFβRI) kinase domain, in combination with capecitabine in refractory mCRC patients with activated TGF-β signature (NCT03470350). A new anti-PD-L1/TGFβRII fusion protein (M7824) is under assessment in a phase Ib/II trial for the treatment of CMS4 or MSI mCRC (NCT03436563).

### 2.8. Epigenetic Alterations

Epigenetic modification, including DNA hypermethylation, is a naturally reversible process regulating gene expression. Abnormal DNA methylation has emerged as a crucial factor for the pathogenesis of several tumor types, including CRC. The 06-metyl-guanine-DNA-methyltransferase (*MGMT*) promoter methylation is an epigenetic silencer, which is an early and frequent event in CRC tumorigenesis. Hypermethylation of gene promoters can be identified in almost 17% of CRC, which frequently have *BRAF* mutations. This epigenetic change involves mainly cytosine-phosphate-guanine (CpG) islands, which is preferentially localized in the promoter of many genes, especially housekeeping and some tissue-specific genes, and leads to subsequent repression of tumor suppressor genes [142,143,144,145,146,147]. 

Calegari et al. conducted a phase II study in pretreated mCRC patients, with *MGMT* promoter methylation showing a modest activity of temozolomide (TMZ) [148]. A recent trial by Pietrantonio et al. evaluated if second-line therapy with capecitabine and TMZ (CAPTEM, arm A) was superior to FOLFIRI (arm B) in patients with *RAS* mutant *MGMT*-methylated mCRC after failure of an oxaliplatin-based regimen. After a median follow-up of 30.5 months, PFS and OS were 3.5 and 9.5 in arm A vs. 3.5 and 10.6 in arm B, respectively, showing the lack of superiority of arm A. Grade ≥3 TRAEs were more frequent in arm B versus A (47.6% vs. 16.3%), while QOL was significantly worse in arm B. In addition, patients with positive MGMT expression as assessed by IHC did not benefit from CAPTEM, so further investigations are required to define who can benefit from the treatment with TMZ [149]. 

A non-randomized phase II trial evaluated 6 cycles of TMZ and irinotecan (TEMIRI) followed by TMZ maintenance in irinotecan-sensitive (irinotecan-free interval >3 months), *MGMT*-methylated or MSS-pretreated mCRC patients. ORR, the primary endpoint, was reached as a result of six patients achieving a PR. Treatment was well tolerated; only 16% of the patients had ≥ grade 3 AEs, and the most common were neutropenia (8%) and diarrhea (4%). An exploratory translational analysis concluded that patients whose cancer was IHC-MGMT-positive were non-responders, while patients with MGMT-negative or -low tumors had a significantly longer mPFS than others (6.9 vs. 2.0 months) and a non-significant trend for longer mOS. For this reason, TEMIRI might be considered an active option in pretreated, irinotecan-sensitive mCRC patients with *MGMT* methylation [150]. 

Currently, the “NIVOLUMAB Plus IPILIMUMAB and TEMOZOLOMIDE in Microsatellite Stable, MGMT Silenced Metastatic Colorectal CancerAYA” MAYA trial (NCT03832621) is enrolling patients with MSS, *MGMT*-silenced mCRC. In the first phase, patients receive single agent TMZ for two cycles; in absence of PD, they go through the second phase, in which they are administered a combination treatment with TMZ, nivolumab, and ipilimumab. The primary efficacy endpoint is the 8-month PFS rate. Enrolment will stop in February 2022. “Pembrolizumab in MMR-Proficient Metastatic Colorectal Cancer Pharmacologically Primed to Trigger Dynamic Hypermutation Status” ARETHUSA study (NCT03519412) is a 2-cohort phase II trial with three different phases. In the “screening” phase, 348 mCRC *RAS* mutant patients are tested for MMR status. Then, dMMR patients proceed directly to the “trial” phase for immediate pembrolizumab treatment. The pMMR patients will be further tested for expression of MGMT by IHC and by promoter methylation analysis. IHC-negative, promoter-methylation-positive pMMR patients enter in the priming phase and are treated with TMZ until PD, when tumor mutational burden will be assessed in tumor biopsies. Patients with >20 mutations/megabase will proceed to the “trial” and will be treated with pembrolizumab. This is based on the assumption that the tumors with acquired resistance to TMZ become hypermutated and potentially sensitive to pembrolizumab. ORR (primary outcome), PFS, OS, and treatment-related toxicities (secondary outcomes) in pMMR pembrolizumab-treated patients will be estimated; the dMMR cohort will be used for comparison. Tissue biopsies, longitudinal blood collection, and stool collection will be used for discovery of predictive molecular biomarkers and assessment of tumor evolution. Enrollment is expected to be concluded in 2020 [151].

## 3. Conclusions

Nowadays, several molecular biomarkers have been identified for the personalized treatment of mCRC patients and extensive efforts have been made to improve tailored therapy. Some of these biomarkers are now consolidated predictive factors for treatment response. *RAS* mutational status determination remains mandatory before starting anti-EGFR treatment with panitumumab and cetuximab, and the new approach of rechallenge based on *RAS/BRAF* status by liquid biopsy is becoming more fascinating [13,14,15,16,17,18,19,20,21,22,23,24,25]. Promising results were obtained for the treatment of HER2+ mCRC [30,31,32,33]. Targeting angiogenesis remains a cornerstone in naïve and pretreated patients, but the identification of validated predictive factors is urgently needed and will hopefully be provided by biologically enriched prospective trials [62]. The BEACON CRC trial has laid the basis for substantial survival improvement for *BRAF^V600E^* mutant patients, and further studies are investigating the combination of BRAF, MEK, and EGFR inhibitors in this category of patients with particularly poor prognosis [92,93,94,95]. Recently, RAS inhibitors showed promising results in *KRAS^G12C^* mutant mCRC, which appeared worthy of further research [88,89,90]. Immunotherapy provided very encouraging data on survival for MSI-H/dMMR mCRC and might be the best area of research, even for MSS *POLE-1* or *POLD1* mutant cancers, due to their high mutational burden [134,135,136,137,138,139]. For rare molecular alterations (*RET*, *NTRK*, *ROS1*, and *ALK* fusions), the results reported in the literature require further research, as well as for patients with *MGMT* methylation [110,111,112,113,114,115,116,117,118,119,120,121,122,123,124,125,148,149,150,151]. 

Currently, in the era of precision medicine, molecular-biology-driven treatment and research in this field, in association with clinical aspects (tumor sidedness, patient characteristics, and aim of therapy), still remain the key to improving the selection of patients and treatment efficacy.

## Figures and Tables

**Table 1:** Anti-epidermal growth factor receptor (EGFR) treatment trials.

Study or Authors	Phase	Treatment	Setting	Primary Endpoint	Results	References
PRIME^1^	III	FOLFOX4^2^–panitumumab vs.^3^ FOLFOX4	First^-^line treatment	PFS^4^	*KRAS^5^ wt^6^:* **mPFS^47^:** 10 months in FOLFOX4–panitumumab arm vs. 8.6 months in FOLFOX4 arm (HR^8^ = 0.80; 95% CI^9^:0.67–0.95; *p* = 0.01) **mOS^10^:** 23.9 months for FOLFOX4–panitumumab arm vs. 19.7 months (95% CI: 17.6–22.7) for FOLFOX4 arm; HR = 0.88; 95% CI: 0.73–1.06; *p* = 0.17) *KRAS mutant:* **mPFS:** 7.4 months in FOLFOX4–panitumumab vs. 9.2 months for FOLFOX4 **mOS:** 15.5 months for FOLFOX4–panitumumab vs. 19.2 months for FOLFOX4 (HR = 1.17, 95% CI: 0.95–1.45; *p* = 0.14)	[13,14]
CRYSTAL^11^	III	FOLFIRI^12^–cetuximab vs. FOLFIRI	First-line treatment	PFS	**mPFS:** 8.9 months for FOLFIRI–cetuximab vs. 8 months for FOLFIRI (HR = 0.85 95% CI: 0.72–0.99; *p* = 0.048) **mOS:** 19.9 months for FOLFIRI–cetuximab vs. 18.6 months for FOLFIRI (HR = 0.93 95% CI: 0.81–1.07; *p* = 0.31) **ORR^13^:** 46.9% for FOLFIRI–cetuximab vs. 38.7 (HR = 1.40 95% CI: 1.12–1.77; *p* = 0.004)	[15]
FIRE-3^14^	III	FOLFIRI–cetuximab vs. FOLFIRI–bevacizumab	First-line treatment *KRAS* exon 2 wt	ORR	*KRAS *exon 2 wt **ORR:** 62.0% FOLFIRI–cetuximab vs. 58.0% FOLFIRI–bevacizumab (OR^15^ = 1.18, 95% CI: 0.85–1.64; *p* = 0.18) **mPFS:** 10.0 months FOLFIRI–cetuximab vs. 10.3 months FOLFIRI–bevacizumab (HR = 1.06, 95% CI: 0.88–1.26; *p* = 0.55) **mOS:** 28.7 months FOLFIRI–cetuximab vs. 25.0 months (HR = 0.77, 95% CI: 0.62–0.96; *p* = 0.017) *All RAS^16^ wt* **ORR:** OR = 1.28, 95% CI: 0.83–1.99; *p* = 0.32) **mPFS:** HR = 0.93, 95% CI: 0.74–1.17; *p* = 0.54) **mOS:** 33.1 months FOLFIRI–cetuximab vs. 25.6 months (HR=0.70, 95% CI: 0.53–0.92; *p* = 0.011)	[16]
PEAK^17^	II	mFOLFOX6^18^–panitumumab vs. mFOLFOX6–bevacizumab	First-line *KRAS* exon 2 wt	PFS	*KRAS exon 2 wt * **mPFS:** 10.9 months in mFOLFOX6–panitumumab vs. 10.1 months in mFOLFOX6–bevacizumab (HR = 0.87;95% CI: 0.65–1.17; *p* = 0.353) **mOS:** 34.2 months in mFOLFOX6–panitumumab vs. 24.3 months in mFOLFOX6–bevacizumab (HR = 0.62;95% CI: 0.44–0.89; *p* = 0.009) **ORR:** 57.8% vs. 53.5% All *RAS* wt: **mPFS:** 13.0 months mFOLFOX6–panitumumab vs. 9.5 months mFOLFOX6–bevacizumab (HR = 0.65;95% CI: 0.44–0.96; *p* = 0.029) **mOS:** 41.3 months in mFOLFOX6–panitumumab vs. 28.9 months in mFOLFOX6–bevacizumab (HR = 0.63;95% CI: 0.39–1.02; *p* = 0.058) **ORR:** 63.6% vs. 60.5%	[17,18]
CALGB/SWOG^19^ 80405	III	FOLFIRI or mFOLFOX6 + cetuximab vs. FOLFIRI or mFOLFOX6 + bevacizumab	First-line *KRAS* exon 2 wt	OS^20^	**mOS:** 29.9 months in cetuximab arm vs. 29.9 months in bevacizumab arm (HR = 0.93;95% CI: 0.78–1.09; *p* = 0.34) **mPFS:** 10.8 months in cetuximab arm vs. 10.4 months in bevacizumab arm (HR = 1.04; 95% CI: 0.91–1.17; *p* = 0.55)	[19]
Santini et al.	II	cetuximab + irinotecan-based therapy	*KRAS* wt mCRC^21^ who had received anti-EGFR^22^ therapy (cetuximab) + FOLFIRI with clinical benefit (confirmed SD^23^ for at least 6 months or clinical response) and after they developed PD^24^, then received a new line of chemotherapy with a break from anti-EGFR therapy (median duration, 6.0 months) and developed PD	ORR	**ORR:** 53.8% (plus 35.9% SD) **mPFS:** 6.6 months	[22]
Siena et al.	Retrospective analysis	rechallenge with an EGFR inhibitor (≥3rd line of therapy)	*RAS* wt mCRC patients treated with anti-EGFR in PRIME and PEAK trials	mOS after rechallenge	mOS after rechallenge: 14.2 months (10.2–17.7) (PRIME: 12.6 moths (9.0–15.3); PEAK: 22.6 months (7.2–42.8)	[23]
CRICKET^25^	II	Rechallenge with cetuximab + irinotecan	*RAS* and *BRAF^26^* wt mCRC; prior first-line irinotecan- and cetuximab-based regimen with at least PR^27^, PFS≥6 months with first-line therapy, and PD within 4 weeks after last dose of cetuximab; prior second-line oxaliplatin- and bevacizumab-based treatment	ORR according to RECIST^28^ 1.1	**ORR:** 21% (95% CI, 10–40%) **DCR^29^:** 54% (95% CI, 36–70%)	[24]
CHRONOS^30^NCT03227926	II	panitumumab	*RAS* wt mCRC patients selected on the basis of *RAS* extended clonal evolution in their plasma (liquid biopsies at specific timepoints)	ORR according to RECIST 1.1	**ongoing**	[25]
TRIPLETE^31^NCT03231722	III	mFOLFOX6 + panitumumab vs. mFOLFOXIRI^32^ + panitumumab	First-line *RAS/BRAF* wt	ORR	**ongoing**	[21]
NCT01198535	I	Cetuximab + RO4929097 (gamma secretase inhibitor)	After first-line treatment	maximum tolerated dose of the combination of cetuximab and RO4929097	**ongoing**	-
NCT03446157	II	Cetuximab + palbociclib	After second-line treatment; refractory *KRAS, NRAS^33^,* and *BRAF* wt; previous anti-EGFR allowed	DCR	**ongoing**	

Note: ^1^PRIME = “Panitumumab Randomized trial In combination with chemotherapy for Metastatic colorectal cancer to determine Efficacy”; ^2^FOLFOX4 = oxaliplatin, 5-fluorouracil, leucovorin, - 4; ^3^vs. = versus; ^4^PFS = progression-free survival; ^5^KRAS = Kirsten RAS oncogene homolog; ^6^wt = wild type; ^47^mPFS = median progression-free survival; ^8^HR = hazard ratio; ^9^CI: = confidence interval; ^10^mOS = median overall survival; ^11^CRYSTAL = “Cetuximab Combined with Irinotecan in First-Line Therapy for Metastatic Colorectal Cancer”; ^12^FOLFIRI = irinotecan, 5-fluorouracil, leucovorin; ^13^ORR = objective response rate; ^14^FIRE-3 = “FOLFIRI plus cetuximab versus FOLFIRI plus bevacizumab as first-line treatment for patients with metastatic colorectal cancer”; ^15^OR = odds ratio; ^16^RAS = rat sarcoma; ^17^PEAK = “Panitumumab Efficacy in Combination With mFOLFOX6 Against Bevacizumab Plus mFOLFOX6 in mCRC Subjects With Wild Type KRAS Tumors”; ^18^mFOLFOX6 = modified oxaliplatin, 5-fluorouracil, leucovorin - 6; ^19^CALGB/SWOG = Cancer and Leukemia Group B/Southwest Oncology Group; ^20^OS = overall survival; ^21^mCRC = metastatic colorectal cancer patients; ^22^anti-EGFR = anti-epidermal growth factor receptor; ^23^SD = stable disease; ^24^PD = progressive disease; ^25^CRICKET = “Cetuximab Rechallenge in Irinotecan-pretreated Mcrc, KRAS, NRAS and BRAF wild type treated in 1st line with anti-EGFR Therapy”; ^26^*BRAF* = v-raf murine sarcoma viral oncogene homolog B1; ^27^PR = partial response; ^28^RECIST = response evaluation criteria in solid tumors; ^29^DCR = disease control rate; ^30^CHRONOS = “Rechallenge With Panitumumab Driven by RAS Dynamic of Resistance”; ^31^TRIPLETE = “Randomized phase III study of triplet mFOLFOXIRI plus panitumumab versus mFOLFOX6 plus panitumumab as initial therapy for unresectable RAS and BRAF wild-type”; ^32^mFOLFOXIRI = modified oxaliplatin, irinotecan, leucovorin, 5-fluorouracil; ^33^*NRAS* = neuroblastoma RAS viral oncogene homolog.

**Table 2:** Anti-human epidermal growth factor receptor 2 (HER2) treatment strategies—phase II trials.

Study	Treatment	Setting	Endpoints	Patients Enrolled	Results	Safety/Toxicity Grade (G)	References
HERACLES^1^ A	Trastuzumab IV^2^ 4 mg^3^/kg^4^ loading dose → 2 mg/kg qw^5^ + lapatinib 1000 mg/daily	*KRAS^6^* exon 2 (codons 12 and 13) wt^7^ e and HER2^48^+ mCRC^9^ refractory to standard of care (including cetuximab or panitumumab)	Primary endpoint: proportion of patients achieving OR^610^	27	OR = 30% (95% CI^711^ 14–50), of which: CR^12^ = 4% (95% CI: 3–11) PR^13^ = 26% (95% CI: 9–43) SD^14^ 44% (95% CI: 25–63)	-22% G3 (fatigue in four patients, skin rash in one patient, increased bilirubin concentration in one patient). -0% G4–G5 -0%SAE^15^	[30]
HERACLES B	Pertuzumab 840 mg loading dose →420 mg q3w^16^ + T-DM1^17^ 3.6 mg/Kg q3w	*RAS^18^/BRAF^19^* wt HER2+ mCRC, PD^20^ after 5-FU^21^, oxaliplatin, irinotecan, and anti-EGFR^22^-containing regimens	Primary endpoint: ORR^23^ Secondary endpoint: PFS^24^	30	-ORR = 10% (95% CI: 0–28) – not significant; SD 70% (95% CI: 50–85); Disease control 80% -mPFS^25^ = 4.8 mos. (95% CI: 3.6–5.8). Higher HER2 IHC^26^ score (3+ vs.^27^ 2+) associated with OR/SD ≥ 4 mos. [*p* = 0.03].	−6.6% G3 thrombocytopenia -G ≤2 events (mainly nausea and fatigue)	[31]
MOUNTAINEER^28^	Tucatinib 300 mg PO^29^ bid^30^ + standard doses of trastuzumab IV q3w	*RAS* wt *HER2+* mCRC, prior treatment with 5-FU, oxaliplatin, irinotecan, and an anti-VEGF^31^	Primary endpoint: ORR	26 → 22 evaluable	-ORR = 55% (CR/PR = 12; SD = 5; PD = 5). -Clinical benefit rate (CR+PR+SD ≥4 months) = 64% -mPFS = 6.2 months (95% CI: 3.5-NE^32^). -mOS^33^ = 17.3 months (95% CI: 12.3-NE) -median duration of response: not reached	−9% G3 −0% G4/5 -most common TRAEs^34^: AST^35^ elevation (48%; all G1), ALT^36^ elevation (30%; all G1), and diarrhea (26%; G1/G2/G3 = 4%/17%/4%).	[32]
TRIUMPH^37^	Tissue and/or ctDNA^38^ confirmed *RAS* wt and *HER2* amplified mCRC, refractory or intolerant to standard chemotherapy, including EGFR blockade	Trastuzumab + pertuzumab q3w	Primary endpoint: ORR, analyzed for two primary populations: tissue-positive and ctDNA-positive	19 → 18 evaluable	-tissue-positive group: ORR = 35% (95% CI: 14–62%); 1 CR and 5 PR -ctDNA positive group: ORR = 33% (95% CI: 12–62%); 1 CR and 4 PR) -mPFS for both groups = 4.0 months (95% CI = 1.4–5.6 months and 1.3–5.6 months, respectively)	-1 patient: G3 decreased ejection fraction -1 patient: G3 infusion related reaction -safety profile consistent with previous reports	[33]

Note: ^1^HERACLES = HER2 Amplification for Colo-rectaL cancer Enhanced Stratification; ^2^IV = intravenously; ^3^mg = milligrams; ^4^kg = kilograms; ^5^qw = once weekly; ^6^KRAS = Kirsten RAS oncogene homolog; ^7^wt = wild type; ^48^HER2+ = human epidermal growth factor receptor 2; ^9^mCRC = metastatic colorectal cancer; ^10^OR = objective response; ^11^CI: = confidence interval; ^12^CR = complete response; ^13^PR = partial response; ^14^SD = stable disease; ^15^SAE = serious adverse event; ^16^q3w = once every 3 weeks; ^17^T-DM1 = trastuzumab emtansine; ^18^RAS = rat sarcoma; ^19^BRAF = v-raf murine sarcoma viral oncogene homolog B1; ^20^PD = progressive disease; ^21^5-FU = 5-fluorouracil; ^22^anti-EGFR = anti-epidermal growth factor receptor; ^23^ORR = objective response rate; ^24^PFS = progression-free survival; ^25^mPFS = median progression-free survival; ^26^IHC = immunohistochemistry; ^27^vs. = versus; ^28^MOUNTAINEER = Phase II, Open Label Study of Tucatinib Combined with Trastuzumab in Patients with HER2+ Metastatic Colorectal Cancer; ^29^PO = per os; ^30^BID = bis in die; ^31^anti-VEGF = anti-vascular endothelial growth factor; ^32^NE = not estimable; ^33^mOS = median overall survival; ^34^TRAEs = treatment related adverse events; ^35^AST = aspartate aminotransferase; ^36^ALT = alanine aminotransferase; ^37^TRIUMPH = Multicenter Phase II study to evaluate efficacy and safety of combination therapy with trastuzumab and pertuzumab in patients with HER2-positive metastatic colorectal cancer; ^38^ctDNA = circulating tumor DNA.

**Table 3:** Bevacizumab trials.

Trial	Phase	Treatment Arms	Setting	Results	Safety/Toxicity Grade (G)	References
Bevacizumab plus Irinotecan, Fluorouracil, and Leucovorin for Metastatic Colorectal Cancer	III	Irinotecan, bolus fluorouracil, and leucovorin (IFL) + bevacizumab vs.^1^ IFL + placebo	First line	Primary endpoint: OS^2^ -mOS^3^: 20.3 months in IFL + bevacizumab group vs. 15.6 months in IFL + placebo group (HR^4^=0.66; *p* < 0.001) -mPFS^5^: 10.6 months in IFL+ bevacizumab vs. 6.2 months in IFL + placebo (HR=0.54; *p* < 0.001) -RR^6^: 44.8% vs. 34.8% (*p* = 0.004)-median duration of the response 10.4 months with IFL+ bevacizumab vs. 7.1 months with IFL + placebo (HR=0.62; *p* = 0.001)	G3 hypertension: 11.0% with IFL + bevacizumab vs. 2.3 with IFL + placebo	[48]
Eastern Cooperative Oncology Group Study E3200	III	FOLFOX4^7^ + bevacizumab vs. FOLFOX4 without bevacizumab vs. bevacizumab alone	After first-line treatment with irinotecan and a fluoropyrimidine	Primary endpoint: OS -mOS: 12.9 months with FOLFOX4 + bevacizumab vs. 10.8 months with FOLFOX4 alone (HR=0.75, *p* = 0.0011) vs. 10.2 months with bevacizumab alone -mPFS: 7.3 months with FOLFOX4 + bevacizumab vs. 4.7 months with FOLFOX4 alone (HR=0.61; *p* = 0.0001) vs. 2.7 months with bevacizumab alone -ORR^8^: 22.7% with FOLFOX4+bevacizumab vs. 8.6% with FOLFOX4 alone vs. 3.3% with bevacizumabalone (*p* = 0.0001 for FOLFOX4 + bevacizumab vs. FOLFOX4).	-any G-3 or 4 AE^9^: 75% with FOLFOX4 + bevacizumab vs. 61% with FOLFOX4 vs. 36% with bevacizumab alone -higher rates of G3 or 4 neuropathy, hypertension, bleeding and vomiting with FOLFOX4 + bevacizumab vs. FOLFOX4	[49]
ML18147^10^	III	Second-line chemotherapy with or without bevacizumab	Second-line treatment after progressing up to 3 months, after discontinuing first-line chemotherapy + bevacizumab	Primary endpoint: OS -mOS: 11.2 months with chemotherapy + bevacizumab vs. 9.8 months with chemotherapy (HR = 0.81, 95% CI^11^: 0.69–0.94; unstratified log-rank test *p* = 0.0062)	-G3-G5 TRAEs^12^: neutropenia (16% vs. 13%), diarrhea (10% vs. 8%) asthenia (6% vs. 4%), bleeding, or hemorrhage (2% vs. <1%), gastrointestinal perforation (2% vs. <1%) and venous thromboembolisms (5% vs. 3%) in the chemotherapy + bevacizumab arm vs. chemotherapy alone arm, respectively. Treatment-related deaths: 4 in the chemotherapy+bevacizumab group vs. 3 in the chemotherapy alone group	[50]
TRIBE^13^	III	FOLFOXIRI^14^ + bevacizumab vs. FOLFIRI^15^ + bevacizumab	First line	Primary endpoint: PFS^16^ -mPFS: 12.1 months vs. 9.7 months, HR 0.75, 95% CI: 0.62–0.90; *p* = 0.003 -mOS: 29.8 months (95% CI: 26·0–34·3) vs. 25·8 months (22.5–29.1)	-G3-G4 neutropenia, diarrhea, stomatitis, and neurotoxicity significantly higher in the experimental group -No significant differences in bevacizumab-related AE - Similar incidence of SAEs^17^ (20.4% vs. 19.7%, *p* = 0.91)	[51,52]
TRIBE-2	III	FOLFOXIRI + bevacizumab →PD^18^→ reintroduction of the same regimen vs. mFOLFOX6^19^ + bevacizumab → PD→ FOLFIRI + bevacizumab	First-line treatment	Primary endpoint: PFS2^20^ -mPFS2^21^ = 19.2 months (95% CI: 17.3–21.4) vs. 16.4 months (15.1–17.5) (HR = 0 = 74, 95% CI: 0.63–0.88; *p* = 0.0005).	First line treatment: -G3-G4 diarrhea (17% vs. 5%), neutropenia (50% vs. 21%), and arterial hypertension (7% vs. 10%); in –SAEs:25% vs. 17% -treatment-related deaths: 8 vs. 4 After first PD: no substantial differences G3-G4 (except neurotoxicity, only reported in the experimental group (5%) -SAEs 15% vs. 12% -3 treatment-related deaths vs. 4	[53]
AVEX^22^	III	Capecitabine + bevacizumab vs. capecitabine	First line	Primary endpoint: PFS -mPFS: 9.1 months (95% CI: 7.3–11.4) vs. 5.1 months (4.2–6.3); HR 0.53 (0.41–0.69); *p* < 0·0001)	-G ≥3: 40% vs. 22% -SAEs: 14% vs. 8% -G ≥3 AE of special interest: HFS^23^ (16% vs. 7%), diarrhea (7% vs. 7%), and venous thromboembolic events (8% vs. 4%) -Any grade AE of special interest for bevacizumab: hemorrhage (25% vs. 7%) -Treatment-related deaths: 5 vs. 4	[55]
TASCO-1^24^	II	Trifluridine/tipiracil + bevacizumab vs. capecitabine + bevacizumab	First line	Primary endpoint: PFS -mPFS: 9.2 months vs. 7.8 months (HR=0.71, 95% CI: 0.48, 1.06)	-SAEs: 54.5%in the experimental arm vs. 57.9% in the control arm; serious febrile neutropenia 3.9% In both arms	[54]
BACCI^25^	II	Capecitabine+bevacizumab+ atezolizumab vs. capecitabine+bevacizumab	Progression on 5-FU^26^, oxaliplatin, irinotecan, bevacizumab, and anti-EGFR^27^ therapy (if *RAS^28^* wt^29^)	Primary endpoint: PFS -mPFS: 3.3 months (2.1–6.2) with capecitabine+bevacizumab + atezolizumab vs. 4.4 months (4.1–6.4) with capecitabine + bevacizumab	G ≥3: hypertension (9% vs. 7%), HFS (6% vs. 4%), and diarrhea (7% vs. 2%).	[57]
SOLSTICE^30^NCT03869892	III	Trifluridine/tipiracil + bevacizumab vs. capecitabine + bevacizumab	First line.Not candidates for intensive chemotherapy	Primary endpoint: PFS	Ongoing	[56]
AtezoTRIBE^31^NCT03721653	II	FOLFOXIRI–bevacizumab-atezolizumab vs. FOLFOXIRI–bevacizumab	First line	Primary endpoint: PFS	Ongoing	-

Note: ^1^vs. = versus; ^2^OS = overall survival; ^3^mOS = median overall survival; ^4^HR = hazard ratio; ^5^mPFS = median progression-free survival; ^6^RR = response rate; ^7^FOLFOX4 = oxaliplatin, 5-fluorouracil, leucovorin – 4; ^8^ORR = objective response rate; ^9^AE = adverse event; ^10^ML18147 = Continuation of bevacizumab after first progression in metastatic colorectal cancer; ^11^CI: = confidence interval; ^12^TRAEs = treatment-related adverse events; ^13^TRIBE = Triplet plus bevacizumab; ^14^FOLFOXIRI = oxaliplatin, irinotecan, leucovorin, 5-fluorouracil; ^15^FOLFIRI = irinotecan, 5-fluorouracil, leucovorin; ^16^PFS = progression free survival; ^17^SAEs = serious adverse events; ^18^PD = progressive disease; ^19^mFOLFOX6 = modified oxaliplatin, 5-fluorouracil, leucovorin – 6; ^20^PFS2 = progression free survival 2; ^21^mPFS2 = median progression-free survival 2; ^22^AVEX = AVastin in the Elderly with Xeloda; ^23^HFS = hand foot syndrome; ^24^TASCO-1 = open-label, randomised, non-comparative phase 2 study evaluating S 95005 (TAS-102) plus bevacizumab and capecitabine plus bevacizumab in patients with previously untreated metastatic COlorectal cancer who are non-eligible for intensive therapy-1 study; ^25^BACCI = Phase II Randomized, Double-Blind, Placebo-Controlled Study of Capecitabine Bevacizumab Plus Atezolizumab Versus Capecitabine Bevacizumab Plus Placebo in Patients With Refractory Metastatic Colorectal Cancer; ^26^5-FU = 5-fluorouracil; ^27^anti-EGFR = anti-epidermal growth factor receptor; ^28^RAS = rat sarcoma; ^29^wt = wild type; ^30^SOLSTICE = An open-label, randomised, phase III Study cOmparing trifLuridine/tipiracil (S 95005) in combination with bevacizumab to capecitabine in combination with bevacizumab in firST-line treatment of patients with metastatIC colorectal cancer who are not candidatE for intensive therapy; ^31^AtezoTRIBE = randomized phase II study of folfoxiri plus Bevacizumab plus atezolizumab versus folfoxiri plus Bevacizumab as first-line treatment of unresectable Metastatic colorectal cancer patients.

**Table 4:** Tyrosine kinase inhibitors (TKIs) for pretreated metastatic colorectal cancer treatment.

TKI	Targets	Phase of the Study	Setting	Results	References
Regorafenib	VEGFR-1^1^, VEGFR-2^2^ VEGFR-3^3^, TIE2^4^, PDGFR, FGFR^6^, KIT^7^, RET^8^, RAF-1^9^, BRAF^10^	III, double-blind, placebo-controlled. CORRECT^11^ trial: regorafenib vs^12^. placebo	mCRC^13^ patients progressing after all approved standard therapies	mOS^14^: 6.4 months with regorafenib vs. 5.0 months with placebo (HR^15^ = 0.77; 95% CI^16^: 0.64–0.94; one-sided *p* = 0·0052)	[68,69]
		III, double-blind, placebo-controlled. CONCUR^17^ trial: regorafenib + BSC^18^ vs. placebo + BSC	mCRC Asian patients with progressive disease after at least two previous treatment lines or who were unable to tolerate standard treatments	mOS: 8.8 months in the regorafenib + BSC group vs. 6.3 months in the placebo group (HR=0.55, 95% CI: 0.40–0.77, one-sided *p* = 0·00016	[70]
		IIIb, open-label. CONSIGN^19^ Study: regorafenib	mCRC patients who progressed after approved standard therapies	Safety profile (primary endpoint) consistent with data from CORRECT and CONCUR trials -mPFS^20^: 2.7 months (95% CI: 2.6–2.7)	[71]
		Ib, open-label, dose-escalation study. Regorafenib + cetuximab	Advanced refractory solid tumors	-Regorafenib 160 mg/day (3 weeks on/1 week off) was declared MTD^21^ in combination with cetuximab -All-grade treatment-emergent adverse events: fatigue (52%), hypophosphatemia (48%), and diarrhea (40%) - PR^22^: 21%	[72]
		Ib, open-label, dose-finding and dose-expansion. REGONIVO/EPOC1603^23^:Regorafenib + nivolumab	Previously treated, advanced gastric cancer or mCRC	- Regorafenib 80 mg + nivolumab had a manageable safety profile -OR^24^ in 38% of patients (including 11 MSS^25^ gastric cancers, 7 MSS mCRC, and 1 MSI-H^26^ mCRC): RR^27^ of 44% in gastric cancer and 29% in MSS mCRC	[73]
Fruquintinib	VEGFR-1,VEGFR-2, VEGFR-3	Ib	Chinese mCRC patients with ≥2 lines of prior therapies	-mPFS:5. 80 months -mOS:8.88 months	[74]
		II, double-blind, placebo-controlled: Fruquintinib + BSC vs. placebo + BSC	Chinese mCRC patients with ≥2 lines of prior therapies	-mPFS: 4.73 months fruquintinib + BSC (95% CI: 2.86–5.59) vs. 0.99 months placebo + BSC; 95% CI: 0.95–1.58); (HR = 0.30; 95% CI:0.15–0.59; *p* < 0.001) -mOS 7.72 vs. 5.52 months (HR = 0.71; 95% CI: 0.38–1.34)	[74]
		III, FRESCO^28^ trial: randomized, double-blind, placebo-controlled, fruquintinib vs. placebo	Chinese mCRC patients progressing after at least 2 prior chemotherapy regimens. Prior anti-VEGFR^29^ therapy not allowed	-mOS: 9.3 months (95% CI, 8.2–10.5) vs. 6.6 months (95% CI, 5.9–8.1; HR = 0.65; 95% CI, 0.51–0.83; *p* < 0.001) -mPFS: 3.7 months (95% CI, 3.7–4.6) vs. 1.8 months [95% CI, 1.8–1.8] months); HR = 0.26 (95% CI, 0.21 to 0.34; *p* < 0.001)	[75]
Axitinib	VEGFR-1, VEGFR-2, VEGFR-3, KIT, PDGFR	II, randomized, double-blinded, placebo-controlled. Maintenance axitinib vs. placebo	mCRC patients that had not progressed after 6–8 months of first-line chemotherapy	mPFS: 4.96 vs. 3.16 months; HR = 0.46; 95% CI: 0.25–0.86; *p* = 0.0116 -mOS = 27.61 vs. 19.99 months; HR = 0.68; 95% CI: 0.31–1.48; *p* = 0.3279)	[76]
		II, single arm Maintenance axitinib	mCRC patients that had not progressed after 4 cycles of first-line mFOLFOX^30^/bevacizumab	mPFS:8.3 months	[77]
Apatinib	VEGFR-2	II	≥Third line	mPFS:3.9 months (95% CI: 2.1–5.9) -mOS:7.9 months (95% CI: 4.6–10.1+)	[78]
Nintedanib	VEGFR, PDGFR, FGFR	I/II; nintedanib+ mFOLFOX-6^31^ vs. bevacizumab + mFOLFOX6	First line	mPFS:10.5 months vs. 15.4 months	[79]
		III; LUME-Colon 1^32^; efficacy and safety of nintedanib + BSC vs. placebo + BSC	mCRC patients after failure of standard therapies (37% pretreated with regorafenib)	-mPFS:1.5 months vs. 1.4 months (HR = 0.58; *p* < 0.0001) -no difference in mOS (6.4 vs. 6.1 months (HR = 1.01; *p* = 0.8659)	[80]

Note: ^1^VEGFR-1 = vascular endothelial growth factor receptor 1; ^2^VEGFR-2 = vascular endothelial growth factor receptor 2; ^3^VEGFR-3 = vascular endothelial growth factor receptor 3; ^4^TIE-2 = tyrosine kinase with immunoglobulin and epidermal growth factor homology domain 2; ^45^PDGFR = platelet-derived growth factor receptor; ^6^FGFR = fibroblast growth factor; ^7^KIT = v-kit Hardy-Zuckerman 4 feline sarcoma viral oncogene homolog; ^8^RET = rearranged during transfection; ^9^RAF-1 = rearranged during transfection; ^10^BRAF = v-raf murine sarcoma viral oncogene homolog B1; ^11^CORRECT = patients with metastatic COloRectal cancer treated with REgorafenib or plaCebo after failure of standard Therapy; ^12^vs. = versus; ^13^mCRC = metastatic colorectal cancer; ^14^mOS = median overall survival; ^15^HR = hazard ratio; ^16^CI: = confidence interval; ^17^CONCUR = Asian Subjects With Metastatic Colorectal Cancer Treated With Regorafenib or Placebo After Failure of Standard Therapy; ^18^BSC = best supportive care; ^19^CONSIGN = Regorafenib for Patients With Metastatic Colorectal Cancer Who Progressed After Standard Therapy; ^20^mPFS = median progression-free survival; ^21^MTD = maximum tolerated dose; ^22^PR = partial response; ^23^REGONIVO/EPOC1603 = Regorafenib + Nivolumab in gastric and colorectal cancer; ^24^OR = objective response; ^25^MSS = microsatellite-stable; ^26^MSI-H = microsatellite instability-high; ^27^RR = response rate; ^28^FRESCO = Fruquintinib Efficacy and Safety in 3+ Line Colorectal Cancer Patients; ^29^VEGFR = vascular endothelial growth factor; ^30^mFOLFOX = modified oxaliplatin, 5-fluorouracil, leucovorin; ^31^mFOLFOX6 = modified oxaliplatin, 5-fluorouracil, leucovorin - 6; ^32^LUME-Colon 1 = A Randomized, Double-blind, Placebo-controlled Phase III Trial of Nintedanib plus Best Supportive Care (BSC) versus Placebo plus BSC in Patients with Advanced Colorectal Cancer Refractory to Standard Treatment.

**Table 5:** Clinical trials targeting the mitogen-activated protein kinase (MAPK) pathway.

Study	Phase	Treatment Arms	Setting	Results	References
NCT03600883	I	AMG 510 (KRAS^G12C 1^ inhibitor)	Locally-advanced/metastatic *KRAS^G12C^* mutant solid tumors	-SD^2^ in 4/19 mCRC^3^ patients –Good tolerability with no dose limiting toxicities at studied doses **Ongoing**	[88,89]
NCT03785249	I/II	MRTX849 (KRAS^G12C^ inhibitor)	Advanced solid tumors with *KRAS^G12C^* mutation	PR^34^ in 1 mCRC patient **Ongoing**	[90]
NCT02928224 BEACON CRC^5^	III	Encorafenib + binimetinib + cetuximab (triplet therapy group) vs.^46^ encorafenib + cetuximab (doublet therapy group) vs. investigators’ choice of either cetuximab and irinotecan or cetuximab and FOLFIRI^7^	*BRAF^V600E^*^8^ mutant mCRC with disease progression after one or two previous regimens	-mOS^9^: 9.0 months in the triplet arm vs. 5.4 months in the control arm (HR^10^ = 0.52; 95% CI^11^: 0.39–0.70; *p* < 0.001); 8.4 months in the doublet arm (HR vs. control, 0.60; 95% CI: 0.45 to 0.79; *p* < 0.001) -RR^12^: 26% in the triplet group and 2% in the control group (*p* < 0.001) - Grade ≥3 TRAEs^13^: 58% of patients in the triplet group, 50% of patients in the doublet therapy group and in 61% in the control group -Reduction of 44% in the risk of quality of life deterioration in patients treated in the experimental arms; no difference in quality of life between triplet and doublet arms	[92,93]
NCT01072175	I/II	Dabrafenib + trametinib	*BRAF^V600E^* or *BRAF^V600k^* ^14^ mutant mCRC; all lines	-CR^15^: 2%, with duration of response of 36 months -SD^16^: 56%	[94]
NCT01750918	I	Dabrafenib + panitumumab vs. dabrafenib + panitumumab + trametinib vs. trametinib + panitumumab	-*BRAF^V600E^* mutant mCRC and mCRC with secondary resistance to prior anti-EGFR^17^ therapy.	RR: 10% for dabrafenib + panitumumab, 21% for dabrafenib + panitumumab + trametinib, and 0% for trametinib+panitumumab	[95]
NCT03693170 ANCHOR –CRC^18^ Study	II	Encorafenib + binimetinib + cetuximab	First-line *BRAF*^19^ mutant mCRC	**Ongoing**	-
NCT04044430	I/II	Encorafenib + binimetinib + nivolumab	MSS^20^ *BRAF^V600E^* mutant mCRC; prior treatment with at least one systemic chemotherapy regimen for mCRC, or recurrence or progression with development of unresectable or metastatic disease within 6 months of adjuvant chemotherapy for resected colorectal cancer	**Ongoing**	-
NCT03727763	II	FOLFIRI + vemurafenib (anti-BRAF) + cetuximab	Advanced *BRAF* mutant mCRC	**Ongoing**	-
NCT03981614	II	Binimetinib + palbociclib vs. trifluridine/tipiracil	*KRAS*^21^ and *NRAS*^22^ mutant mCRC	**Ongoing**	-
NCT02906059	I	Irinotecan + AZD1775 (adavosertib; selective WEE1^23^-inhibitor)	Second-line *RAS* or *BRAF* mutant mCRC	**Ongoing**	-

Note: ^1^KRAS^G12C^ = Kirsten RAS oncogene homolog G12C; ^2^SD = stable disease; ^3^mCRC = metastatic colorectal cancer; ^4^PR = partial response; ^5^BEACON CRC = Binimetinib, Encorafenib, and Cetuximab Combined to Treat BRAF-Mutant Colorectal Cancer; ^46^vs. = versus; ^7^FOLFIRI = irinotecan, 5-fluorouracil, leucovorin; ^8^BRAF^V600E^ = v-raf murine sarcoma viral oncogene homolog B1 V600E; ^9^mOS = median overall survival; ^10^HR = hazard ration; ^11^CI: = confidence interval; ^12^RR = response rate; ^13^TRAEs = treatment related adverse events; ^14^BRAF^V600k^ = v-raf murine sarcoma viral oncogene homolog B1 V600k; ^15^CR = complete response; ^16^SD = stable disease; ^17^anti-EGFR: anti-epidermal growth factor receptor; ^18^ANCHOR CRC = Encorafenib, Binimetinib and Cetuximab in Subjects With Previously Untreated BRAF-mutant ColoRectal Cancer; ^19^BRAF = v-raf murine sarcoma viral oncogene homolog B1; ^20^MSS = microsatellite-stable; ^21^KRAS = Kirsten RAS oncogene homolog; ^22^NRAS = neuroblastoma RAS viral oncogene homolog; ^23^WEE1 = WEE1 G2 checkpoint kinase.

**Table 6:** Immunotherapy trials involving metastatic colorectal cancer patients.

Study	Study Type	Treatment	Setting	Primary Endpoint	Results
KEYNOTE-164 ^1^NCT02460198 [134]	Phase II, open-Label	Pembrolizumab (anti-PD-1)^2^	Previously treated MSI-H/dMMR^3^ mCRC^4^: -Cohort A: ≥2 prior lines of standard therapy, including fluoropyrimidine, oxaliplatin, and irinotecan ± anti-VEGF^45^/EGFR^6^ monoclonal antibodies -Cohort B: ≥1 prior line of therapy	ORR^7^ by RECIST^8^ version 1.1 by independent central review	-Cohort A: *ORR: 33% (95% CI^9^: 21–46%); median duration of response not reached *mPFS^10^: 2.3 months (95% CI, 2.1–8.1 months) *mOS^11^: 31.4 months (95% CI, 21.4 months-not reached) -Cohort B: *ORR: 33% (95% CI: 22–46%); median duration of response not reached *mPFS: 4.1 months (95% CI, 2.1–18.9 months) *mOS: not reached (95% CI, 19.2 months—not reached)
CheckMate-142^12^ trial NCT02060188 [135,136,137]	Phase II, open-label, non-randomized, multicohort	Nivolumab (anti-PD-1)	MSI-H/dMMR recurrent/mCRC progressed on/after, or intolerant of at least one previous line of treatment, including fluoropyrimidine and oxaliplatin or irinotecan	ORR - investigator-assessed per RECIST 1.1	*ORR: 31.1% (95% CI: 20.8–42·9%) of patients * disease control for ≥12 weeks: 68.9% of patients (95% CI: 57.1–79.2%) *median duration of response: not yet reached
		Nivolumab + ipilimumab (anti-CTLA-4^13^)	First line MSI-H/dMMR mCRC		*ORR: 60%
2 – NIMBLe^14^ study NCT0346195	Phase II, open-label, randomized, non-comparative trial	Nivolumab vs.^15^ nivolumab+ipilimumab	Advanced hypermutated solid tumors with POLE^16^ or POLD1^17^ mutations progressing after at least 1 standard cancer therapy	ORR by RECIST 1.1	**Ongoing**
NCT03428802	Basket, open-label trial	Pembrolizumab	Metastatic, recurrent. or locally advanced cancer and genomic instability, including POLE and POLD1 mutations	RR^18^	**Ongoing**
NCT03150706	Phase II, open-label	Avelumab (anti-PD-L1)^19^	MSI-H/dMMR or POLE mutant mCRC progressed after at least first-line systemic chemotherapy	Serum CEA^20^, TSH^21^, T3^22^, free T4^23^, EKG^24^, CT^25^ (or MRI^26^) scans of evaluable/measurable lesions by RECIST 1.1	**Ongoing**
NCT03435107	Phase II, open-label	Durvalumab (anti-PD-L1)	MSI-H/dMMR or POLE mutant mCRC progressed after at least first-line systemic chemotherapy for metastatic setting (or within 6 months after completion of adjuvant chemotherapy)	ORR by RECIST 1.1	**Ongoing**

Note: ^1^KEYNOTE-164 = A Phase II Study of Pembrolizumab (MK-3475) as Monotherapy in Subjects with Previously Treated Locally Advanced Unresectable or Metastatic (Stage IV) Mismatched Repair Deficient or Microsatellite Instability-High Colorectal Carcinoma; ^2^anti-PD1 = anti-programmed cell death protein 1; ^3^MSI-H/dMMR = microsatellite instability-high/defective mismatch repair; ^4^mCRC = metastatic colorectal cancer; ^45^anti-VEGF = anti-vascular endothelial growth factor; ^6^EGFR = epidermal growth factor receptor; ^7^ORR = objective response rate; ^8^RECIST = response evaluation criteria in solid tumors; ^9^CI: = confidence interval; ^10^mPFS = median progression-free survival; ^11^mOS = median overall survival; ^12^CheckMate-142 = Nivolumab in Patients With Metastatic DNA Mismatch Repair-Deficient or Microsatellite Instability-High Colorectal Cancer; ^13^anti-CTLA4 = anti -cytotoxic T-lymphocyte antigen-4; ^14^NIMBLe = Nivolumab Ipilimumab in Patients With hyperMutated Cancers Detected in Blood; ^15^vs. = versus; ^16^POLE = DNA polymerase epsilon, catalytic subunit; ^17^POLD1 = DNA polymerase delta 1, catalytic subunit; ^18^RR = response rate; ^19^anti-PD-L1 = anti-programmed death-ligand 1; ^20^CEA = carcinoembryonic antigen; ^21^TSH = thyroid-stimulating hormone; ^22^T3 = triiodothyronine; ^23^T4 = thyroxine; ^24^EKG = electrocardiogram; ^25^CT = computed tomography; ^26^MRI = magnetic resonance imaging.
